# Use of the ICD-10 vision codes to study ocular conditions in Medicare beneficiaries with stroke

**DOI:** 10.1186/s12913-020-05484-z

**Published:** 2020-07-08

**Authors:** Kimberly P. Hreha, Steve R. Fisher, Timothy A. Reistetter, Kenneth Ottenbacher, Allen Haas, Chih-Ying Li, Joshua R. Ehrlich, Diane B. Whitaker, Heather E. Whitson

**Affiliations:** 1 Division of Rehabilitation Sciences, University of Texas Medical Branch, School of Health Professions, 301 University Blvd., Galveston, TX 77555 USA; 2 Physical Therapy Department, University of Texas Medical Branch, School of Health Professions, 301 University Blvd., Galveston, TX 77555 USA; 3 Department of Occupational Therapy, School of Health Professions, University of Texas, 7703 Floyd Curl Drive, San Antonio, TX 78229 USA; 4 Department of Preventive Medicine and Population Health, University of Texas Medical Branch, 301 University Blvd., Galveston, TX 77555 USA; 5 Occupational Therapy Department, University of Texas Medical Branch, School of Health Professions, 301 University Blvd., Galveston, TX 77555 USA; 6 Department of Ophthalmology and Visual Sciences, Kellogg Eye Center, University of Michigan, 100 Wall Street, Ann Arbor, MI 48105 USA; 7 Department of Ophthalmology, Duke University School of Medicine, 2351 Erwin Rd, Durham, NC 27705 USA; 8 Department of Medicine, Duke University School of Medicine, Durham, NC USA; 9 Center for the Study of Aging and Human Disease, Duke University School of Medicine, Durham, NC USA; 10 Geriatrics Research Education and Clinical Center, Durham VA Medical Center, 8 Searle Center Drive, Durham, NC 27710 USA

**Keywords:** Vision, Impairment, Ocular conditions, Stroke, Comorbidity, Medical coding

## Abstract

**Background:**

Ocular conditions are common following stroke and frequently occur in combination with pre-existing ophthalmologic disease. The Medicare International Statistical Classification of Diseases and Related Health Problems (ICD-10) coding system for identifying vision related health conditions provides a much higher level of detail for coding these complex scenarios than the previous ICD-9 system. While this new coding system has advantages for clinical care and billing, the degree to which providers and researchers are utilizing the expanded code structure is unknown. The purpose of this study was to describe the use of ICD-10 vision codes in a large cohort of stroke survivors.

**Methods:**

Retrospective cohort design to study national 100% Medicare claims files from 2015 through 2017. Descriptive data analyses were conducted using all available ICD-10 vision codes for beneficiaries who had an acute care stay because of a new stroke. The outcome of interest was ≥1 ICD-10 visual code recorded in the claims chart.

**Results:**

The cohort (*n* = 269,314) was mostly female (57.1%) with ischemic stroke (87.8%). Approximately 15% were coded as having one or more ocular condition. Unspecified glaucoma was the most frequently used code among men (2.83%), those over 85+ (4.80%) and black beneficiaries (4.12%). Multiple vision codes were used in few patients (0.6%). Less than 3% of those in the oldest group (85+ years) had two or more vision codes in their claims.

**Conclusions:**

Ocular comorbidity was present in a portion of this cohort of stroke survivors, however the vision codes used to describe impairments in this population were few and lacked specificity. Future studies should compare ophthalmic examination results with billing codes to characterize the type and frequency of ocular comorbidity. It important to understand how the use of ICD-10 vision codes impacts clinical decision making, recovery, and outcomes.

## Background

Stroke is the leading cause of disability in the United States (U.S.) and worldwide [1]. One major disability resulting from stroke is ocular conditions and vision impairment [1]. For acute stroke survivors, the prevalence of vision impairments has been reported to range from 65 to 92% [2–4] and the incidence of new visual impairments, at stroke onset was 59.6% [3]. Approximately 60% of people with chronic stroke report vision deficits including ocular deficits [5]. Stroke or neurologically-related vision or eye problems typically include conditions such as visual field loss, ptosis, strabismus, or oculomotor impairments [4]. However stroke survivors may also have an elevated risk of other types of vision impairments that share common risk factors with stroke, such as diabetic retinopathy, since diabetes is a common risk factor for stroke and vision impairment [6]. Additionally, stroke survivors may have comorbid ophthalmologic diseases, particularly conditions such as glaucoma, age-related macular degeneration, and cataracts, that are common among older adults [3, 7].

Healthcare providers use hospital medical coding to classify diagnoses and reasons for medical visits. The Medicare International Statistical Classification of Diseases and Related Health Problems (ICD-10) codes, which replaced ICD-9 codes on October 1, 2015, is the current coding system used in the U.S. The ICD-10 system brought changes which included: rearrangement of parts of the code book, alphanumeric characters used for all codes, modernization and harmonization of the terminology, the use of new codes combining diagnoses and symptoms, as well as a significant increase in the specificity of the reporting. Differences between ICD-9 and ICD-10 are highlighted by number alone-- there are over 71,000 diagnostic codes in the ICD-10 versus approximately 13,000 ICD-9 codes [8]. The level of detail present in the ICD-10 codes requires much greater specificity in medical documentation and code selection.

While the granularity of the new coding system has advantages for clinical care, the added complexity may create challenges for population health research and even hospital billing providers. For example, it may be more time intensive for providers to search through all 71,000 codes to determine the most appropriate code to use for every patient admitted for medical services. The objective of this study was to determine the patterns of use of ICD-10 codes to identify vision or eye problems in a national stroke cohort. We hypothesized that the range of codes used in a stroke cohort would be limited and biased toward neuro-ophthalmic diagnoses, because of the inciting neurological event. We further conjectured that stroke survivors would be likely to have multiple eye and vision codes, including non-neurologic codes, since age and certain medical conditions are common risk factors for eye disease and stroke.

## Methods

We employed a retrospective cohort design and nationwide 100% Medicare claims data between October 1, 2015 and December 31, 2017. This study use data from a parent study that is examining rehabilitation services and was approved by the Institutional Review Board at the University of Texas Medical Branch*.* This organization also holds a data usage agreement with the Centers for Medicare and Medicaid Services.

### Study sample

**Fig. 1:**
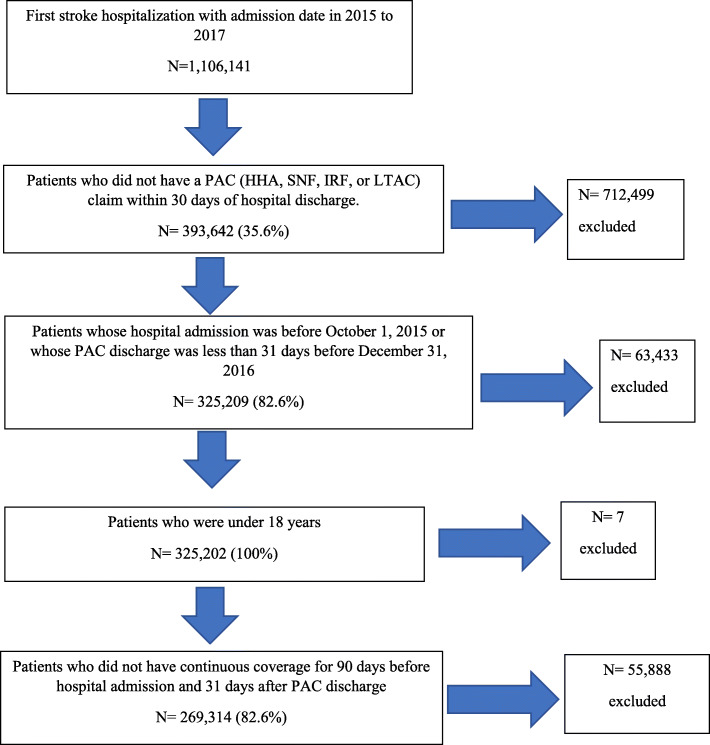
Consort Diagram of cohort selection. Note: Values in parentheses are the percentage of the previous step remaining; PAC: Post Acute Care; HHA: Home Health Agency; SNF: Skilled Nursing Facility; IRF: Inpatient Rehabilitation Facility; LTAC: Long Term Assistive Care

The population of interest was participants who had an acute care stay because of a primary diagnosis of stroke based on the Rehabilitation Impairment Category codes 01.1, 01.2, 01.3, 01.4, 01.9 who also had an ICD-10 vision/eye code recorded at least once in their claims file (*n* = 1,106,141). Based on our exclusion criteria, we removed beneficiaries without a post-acute care claim within 30 days of hospital discharge, to be able to determine whether the stroke codes used are incident or prevalent diagnoses (*n* = 712,499). Then we removed those whose hospital admission or post-acute care discharge was outside our study window and those under age 18 (*n* = 63,433). Finally, we removed those without continuous Medicare coverage for 90 days before the hospital admission and 31 days after post-acute care discharge, which allow us to track patients continuously (*n* = 55,888). See Fig. 1 for a diagram of our study sample selection.

### Study variables

#### Ocular conditions

The 203 main vision and eye related ICD-10 main codes were used to determine any eye disorder or vision impairment present in the patient’s medical record (www.findacode.com). For example, the main code for glaucoma is H40.X. Under the main code are sub-codes. H40.0 through H40.8 are sub-codes for glaucoma, which include information such as type of glaucoma and which eye is affected. These sub-codes provide very detailed information, such as if the glaucoma is secondary to another ocular disorder and the stage of the disease.

#### Demographic and clinical factors

Patient characteristics included: 1) age at admission (categorized into the following groups: 18–49, 50–64, 65–74, 75–84, and 85+ years); 2) race/ethnicity (non-Hispanic white, non-Hispanic black, Hispanic, other); 3) type of stroke (hemorrhagic, ischemic); 4) gender (male, female); 5) Medicaid eligibility (yes, no), used as a proxy for socioeconomic status [9]; and 6) the comorbidities listed in the Elixhauser Comorbidity Index [10]. Clinical factors included: 1) length of stay in acute care (1–2, 3, 4, 5, 6–7, 8+ days); 2) post-acute care facility (home health, inpatient rehabilitation, long term acute care, or skilled nursing); and 3) stays in the intensive care unit/coronary care unit (yes, no), used as a proxy for stroke severity [11].

### Data analysis

We provided counts and percentages of the sample demographic variables. The codes that were identified as present in the medical chart were codes billed during the post-acute care stays. The five most common eye and vision codes were identified for the entire cohort; we then calculated the prevalence of these codes by demographic and clinical variables. We also calculated the most common eye and vision codes for individuals with each of the 31 medical conditions in the Elixhauser Comorbidity Index [10]. Lastly, frequencies and percentages of the most common codes were stratified by age group. Data management was completed before analysis because it required merging of multiple datasets. The analyses were performed with SAS statistical software Version 9.4 (SAS, Inc., Cary, NC).

## Results

Our initial dataset had 1,106,141 beneficiaries who both sustained a new stroke and had an ocular condition. The final cohort, after application of selection criteria, included 269,314 individuals (Fig. 1). Table 1 presents the characteristics of the study sample. More than half were female (57.1%) with a higher prevalence of ischemic stroke compared to hemorrhagic (87.8% versus 12.2%, respectively). The most common racial/ethnic group was non-Hispanic white (76.2%) and the most common age group was 75–84 years (33.4%). In all, 14.8% of Medicare beneficiaries with stroke were identified as having at least one eye or vision diagnosis upon admission to acute care. Table 1 also describes the percentage with eye and vision codes for each demographic variable. There were roughly an equal percentage of men and women with these codes. Approximately 23% discharged to inpatient rehabilitation had one or more eye or vision diagnosis.

**Table 1:** Cohort Demographics

Demographic	N (%)	Percentage with Vision Code (95% CI)
**Overall**	269,314	14.8% (14.7, 14.9)
**Gender**		
Male	115,570 (42.9%)	14.9% (14.7, 15.1)
Female	153,744 (57.1%)	14.7% (14.6, 14.9)
**Age**		
18–49	2997 (1.1%)	16.5% (15.2, 17.9)
50–64	19,716 (7.3%)	14.5% (14.0, 15.0)
65–74	71,697 (26.6%)	14.2% (14.0, 14.5)
75–84	90,078 (33.4%)	14.2% (14.0, 14.4)
85 +	84,826 (31.5%)	16.0% (15.7, 16.2)
**Race/Ethnicity**		
Non-Hispanic White	205,306 (76.2%)	15.0% (14.9, 15.2)
Non-Hispanic Black	37,832 (14.0%)	14.1% (13.7, 14.4)
Hispanic	15,239 (5.7%)	14.2% (13.7, 14.8)
Other	10,937 (4.1%)	13.9% (13.2, 14.5)
**Original Entitlement**		
Age	216,115 (80.2%)	15.0% (14.8, 15.1)
Disability	50,591 (18.8%)	14.0% (13.7, 14.3)
End Stage Renal Disease (ESRD)	1255 (0.5%)	19.7% (17.5, 21.9)
Disability and ESRD	1353 (0.5%)	19.6% (17.5, 21.7)
**Dual Medicare/Medicaid Eligible**		
No	194,910 (72.4%)	15.6% (15.4, 15.7)
Yes	74,404 (27.6%)	12.8% (12.6, 13.1)
**Intensive Care Unit days**		
0	119,053 (44.2%)	14.8% (14.6, 15.0)
1–2	53,501 (19.9%)	15.2% (14.9, 15.5)
3–4	52,682 (19.6%)	15.4% (15.1, 15.7)
5+	44,078 (16.4%)	13.8% (13.5, 14.1)
**Length of Acute Stay**		
1–2	55,391 (20.6%)	15.8% (15.5, 16.1)
3	64,601 (24.0%)	15.2% (14.9, 15.5)
4	44,591 (16.6%)	15.5% (15.2, 15.8)
5	30,335 (11.3%)	15.1% (14.7, 15.5)
6–7	34,873 (12.9%)	14.6% (14.2, 15.0)
8+	39,523 (14.7%)	12.0% (11.6, 12.3)
**Post-Acute Care Type**		
Home Health Agency	56,954 (21.1%)	10.5% (10.3, 10.8)
Inpatient Rehabilitation Facility	101,278 (37.6%)	22.8% (22.5, 23.0)
Long Term Acute Care	2689 (1.0%)	10.0% (8.9, 11.1)
Skilled Nursing Facility	108,393 (40.2%)	9.7% (9.6, 9.9)
**Stroke Type**		
Hemorrhagic	32,747 (12.2%)	14.6% (14.2, 14.9)
Ischemic	236,546 (87.8%)	14.9% (14.7, 15.0)

Table 2 presents the most used ICD-10 vision codes by demographics, which were: unspecified glaucoma, unspecified age-related macular degeneration, diplopia, other visual disturbance, and homonymous visual field defect. Unspecified glaucoma was the code most used in the group of 85+ years (4.8%), females (3.4%), and non-Hispanic blacks (4.1%). Glaucoma was also the most common condition identified in the inpatient rehabilitation group (4.2%). 192 of the 203 vision codes were never used in this cohort. Five of the codes were used with more than 1% of individuals in this dataset.

**Table 2:** Most Common ICD-10 Vision Codes by Demographics

Demographic	H409 (unspecified glaucoma)	H3530 (unspecified macular degeneration)	H532 (diplopia)	H538 (other visual disturbances)	H53462 (homonymous bilateral field cut, left side)
**Gender**					
Male	3272 (2.83%)	1409 (1.22%)	1904 (1.65%)	1269 (1.10%)	1020 (0.88%)
Female	5176 (3.37%)	3380 (2.20%)	1797 (1.17%)	1470 (0.96%)	1219 (0.79%)
**Age**					
18–49	26 (0.87%)	*	53 (1.77%)	67 (2.24%)	20 (0.67%)
50–64	239 (1.21%)	41 (0.21%)	354 (1.80%)	289 (1.47%)	163 (0.83%)
65–74	1353 (1.89%)	307 (0.43%)	1490 (2.08%)	957 (1.33%)	734 (1.02%)
75–84	2762 (3.07%)	1326 (1.47%)	1222 (1.36%)	865 (0.96%)	775 (0.86%)
85 +	4068 (4.80%)	3114 (3.67%)	582 (0.69%)	561 (0.66%)	547 (0.64%)
**Race/Ethnicity**					
Non-Hispanic White	6135 (2.99%)	4555 (2.22%)	2946 (1.43%)	2058 (1.00%)	1847 (0.90%)
Non-Hispanic Black	1559 (4.12%)	78 (0.21%)	422 (1.12%)	396 (1.05%)	207 (0.55%)
Hispanic	417 (2.74%)	75 (0.49%)	188 (1.23%)	154 (1.01%)	99 (0.65%)
Other	337 (3.08%)	81 (0.74%)	145 (1.33%)	131 (1.20%)	86 (0.79%)
**Reason why on Medicare**					
Old age	7399 (3.42%)	4471 (2.07%)	2893 (1.34%)	2108 (0.98%)	1848 (0.86%)
Disability Insurance	992 (1.96%)	306 (0.60%)	781 (1.54%)	600 (1.19%)	387 (0.76%)
End Stage Renal Disease (ESRD)	26 (2.07%)	*	11 (0.88%)	16 (1.27%)	*
Disability and ESRD	31 (2.29%)	*	16 (1.18%)	15 (1.11%)	*
**Dual Medicare/Medicaid Eligible**					
No	6501 (3.34%)	4055 (2.08%)	3031 (1.56%)	2125 (1.09%)	1781 (0.91%)
Yes	1947 (2.62%)	734 (0.99%)	670 (0.90%)	614 (0.83%)	458 (0.62%)
**Intensive Care Unit days**					
0	3664 (3.08%)	2337 (1.96%)	1639 (1.38%)	1199 (1.01%)	899 (0.76%)
1–2	1692 (3.16%)	1019 (1.90%)	812 (1.52%)	576 (1.08%)	490 (0.92%)
3–4	1736 (3.30%)	917 (1.74%)	731 (1.39%)	581 (1.10%)	473 (0.90%)
5+	1356 (3.08%)	516 (1.17%)	519 (1.18%)	383 (0.87%)	377 (0.86%)
**Length of Acute Stay**					
1–2	1676 (3.03%)	1173 (2.12%)	980 (1.77%)	715 (1.29%)	484 (0.87%)
3	2155 (3.34%)	1315 (2.04%)	917 (1.42%)	663 (1.03%)	493 (0.76%)
4	1503 (3.37%)	872 (1.96%)	632 (1.42%)	493 (1.11%)	391 (0.88%)
5	967 (3.19%)	541 (1.78%)	398 (1.31%)	269 (0.89%)	269 (0.89%)
6–7	1077 (3.09%)	536 (1.54%)	458 (1.31%)	327 (0.94%)	307 (0.88%)
8+	1070 (2.71%)	352 (0.89%)	316 (0.80%)	272 (0.69%)	295 (0.75%)
**Post-Acute Care Type**					
Home Health	966 (1.70%)	849 (1.49%)	466 (0.82%)	429 (0.75%)	255 (0.45%)
Inpatient Rehabilitation Facility	4258 (4.20%)	2189 (2.16%)	2849 (2.81%)	2136 (2.11%)	1752 (1.73%)
Long Term Acute Care	58 (2.16%)	15 (0.56%)	15 (0.56%)	16 (0.60%)	12 (0.45%)
Skilled Nursing Facility	3166 (2.92%)	1736 (1.60%)	371 (0.34%)	158 (0.15%)	220 (0.20%)

The number of codes per patient by age group is shown in Table 3. One code (glaucoma) was found most frequently in the 85+ group (12.8%). Two as well as three or more codes were used in all groups, but overall the application of multiple eye and vision codes was infrequent. For example, 2.6% of those in the oldest group (85+ years) had 2 eye or vision codes noted in their claims.

**Table 3:** Number of Vision Impairment Codes per Patient by Age Group

Vision Codes	18–49	50–64	65–74	75–84	85+
1	377 (12.6%)	2263 (11.5%)	8237 (11.5%)	10,395 (11.5%)	10,844 (12.8%)
2	88 (2.9%)	458 (2.3%)	1514 (2.1%)	1867 (2.1%)	2196 (2.6%)
3+	31 (1.0%)	142 (0.7%)	456 (0.6%)	537 (0.6%)	496 (0.6%)

Table 4 shows which eye and vision codes were used among stroke survivors with the top 31 Elixhauser comorbidities. Glaucoma is also the most common code among people living with chronic heart failure, valvular disease, anemia, and others. In addition, 21.5% of individuals with any vision code had diabetes, 18% depression, and 15% psychoses.

**Table 4:** Vision Codes by Elixhauser Comorbidity

Comorbidity	N	Any Vision Code	Most Common Code	N (%)	Second Most Common Code	N (%)	Third Most Common Code	N (%)
Chronic Heart Failure	37,627	5969 (15.9%)	H409	1273 (3.4%)	H3530	812 (2.2%)	H532	462 (1.2%)
Valvular Disease	13,807	2950 (21.4%)	H409	657 (4.8%)	H3530	469 (3.4%)	H532	298 (2.2%)
Pulmonary Circulation Disease	1798	279 (15.5%)	H409	67 (3.7%)	H3530	24 (1.3%)	H53462	18 (1.0%)
Peripheral Vascular Disease	18,203	3417 (18.8%)	H409	690 (3.8%)	H3530	440 (2.4%)	H532	307 (1.7%)
Hypertension	207,517	34,413 (16.6%)	H409	7399 (3.6%)	H3530	4106 (2.0%)	H532	3228 (1.6%)
Paralysis	22,930	3277 (14.3%)	H409	642 (2.8%)	H3530	343 (1.5%)	H532	289 (1.3%)
Other Neurological Disorders	14,685	1567 (10.7%)	H409	401 (2.7%)	H3530	225 (1.5%)	H548	94 (0.6%)
Chronic Pulmonary Disease	39,337	6115 (15.5%)	H409	1199 (3.0%)	H3530	803 (2.0%)	H532	576 (1.5%)
Diabetes without Chronic Complications	53,231	7227 (13.6%)	H409	1648 (3.1%)	H3530	721 (1.4%)	H532	700 (1.3%)
Diabetes with Chronic Complications	37,541	8054 (21.5%)	E11319	1628 (4.3%)	H409	1304 (3.5%)	H532	767 (2.0%)
Hypothyroidism	37,092	7022 (18.9%)	H409	1742 (4.7%)	H3530	1094 (2.9%)	H532	541 (1.5%)
Renal Failure	41,135	7504 (18.2%)	H409	1501 (3.6%)	E11319	817 (2.0%)	H3530	785 (1.9%)
Liver Disease	2866	453 (15.8%)	H409	78 (2.7%)	H532	56 (2.0%)	H3530	35 (1.2%)
Peptic Ulcer Disease	1679	332 (19.8%)	H409	76 (4.5%)	H3530	37 (2.2%)	H532	32 (1.9%)
Acquired Immunodeficiency Syndrome	400	44 (11.0%)	H409	13 (3.3%)	*	*	*	*
Lymphoma	1369	239 (17.5%)	H409	38 (2.8%)	H532	30 (2.2%)	H3530	21 (1.5%)
Metastatic Cancer	2268	310 (13.7%)	H409	50 (2.2%)	H3530	31 (1.4%)	H538	28 (1.2%)
Solid Tumor without Metastasis	6626	955 (14.4%)	H409	231 (3.5%)	H3530	122 (1.8%)	H538	65 (1.0%)
Rheumatoid Arthritis	7296	1299 (17.8%)	H409	287 (3.9%)	H3530	153 (2.1%)	H532	112 (1.5%)
Coagulopathy	5790	1150 (19.9%)	H409	225 (3.9%)	H3530	139 (2.4%)	H532	116 (2.0%)
Obesity	18,427	3616 (19.6%)	H409	555 (3.0%)	H532	474 (2.6%)	E11319	350 (1.9%)
Weight Loss	7586	1309 (17.3%)	H409	294 (3.9%)	H3530	153 (2.0%)	H532	84 (1.1%)
Fluid/Electrolyte Disorders	28,909	5929 (20.5%)	H409	1249 (4.3%)	H3530	676 (2.3%)	H532	577 (2.0%)
Chronic Blood Loss Anemia	808	133 (16.5%)	H409	37 (4.6%)	H3530	17 (2.1%)	H532	12 (1.5%)
Deficiency Anemias	33,282	6290 (18.9%)	H409	1476 (4.4%)	H3530	713 (2.1%)	H532	447 (1.3%)
Alcohol Abuse	5570	869 (15.6%)	H532	122 (2.2%)	H409	112 (2.0%)	H538	75 (1.3%)
Drug Abuse	1621	229 (14.1%)	H532	30 (1.9%)	H409	25 (1.5%)	H53462	20 (1.2%)
Psychoses	6744	1014 (15.0%)	H409	210 (3.1%)	H3530	90 (1.3%)	H532	79 (1.2%)
Depression	44,988	8093 (18.0%)	H409	1724 (3.8%)	H3530	992 (2.2%)	H532	726 (1.6%)

*Notes:* H409- unspecified glaucoma; E11319- type 2 diabetes mellitus with unspecified diabetic retinopathy without macular edema; H532- diplopia; H3530- unspecified macular degeneration; H53462- homonymous bilateral field defects, left side; H538- other visual disturbances; H548- legal blindness, as defined in United States of America

## Discussion

In this nationally representative sample of 100% Medicare claims files, the application of eye and vision billing codes in an acute stroke population varied by patient demographics, but the frequency of coding for ocular conditions was less than expected. Overall, only a small number of codes were used to describe the ocular conditions in this cohort, despite the very large number of codes now available in ICD-10. It is possible that these codes represent the only ocular conditions that were present in this cohort. However other studies report that older adults have general multimorbidity, which puts them at risk for ocular conditions [12], as well as a high prevalence of ophthalmologic comorbidity [13].

It is noteworthy that the majority of the patients had just one eye/vision code. About 2 % of the sample had two vision impairment codes and less than 1 % had three or more codes. Based on other reports of high prevalence of ocular conditions in stroke (60%), we speculate that the number of individuals with multiple vision impairments might be under reported for stroke survivors under Medicare coverage, due to the lack of mandated and systematic assessment procedures [14].

The most frequently used single vision billing code was glaucoma. Specifically, the glaucoma codes were most prevalent among non-Hispanic Blacks, which is consistent with prior research reports of non-Hispanic Blacks have higher prevalence of glaucoma [15, 16]. Glaucoma was also more common among females and older individuals in our study, as expected for an ocular disease that is strongly age-related. We hypothesized that age-related eye conditions would be present but not at a rate considerably higher than in the general population since this was a post-acute care cohort without ophthalmology clinic data. Thus, this finding was surprising. One possibility is that these codes were taken from past medical histories which could include previous ophthalmology records and specific terminology. This may also have been the case if certain medications (e.g. eye drops for glaucoma) were continued during the inpatient hospital stay. If a diagnosis is not evident but a patient is taking a specific medication at the time of admission, the medical team may investigate further in order to ensure continuation of the person’s outpatient eye care.

Currently, only two acute-care based studies have reported the prevalence and frequency of multiple vision impairments in stroke survivors [3, 4]. These were reports that used prospective clinical data and were not restricted to Medicare beneficiaries because they were based in the United Kingdom [3, 4]. Unlike this study’s findings (15% of the total group had ocular conditions), the researchers found that approximately 72.8% of individuals admitted for a stroke had abnormal findings on their ophthalmologic assessments [3]. And because the study was prospective, they were able to report on the timing of the vision assessment including that 13% had visual problems that pre-existed the stroke, 27% had both new stroke visual problems and pre-existing vision and ocular impairments and 32% had new onset stroke-related visual conditions [3]. Similarities were found between this study and ours. For example, our study found that the 85+ group had the highest percentage (2.6%) of having two vision codes documented among the group. Rowe et al. reported that the highest percentage of documented visual problems were found among the 80–90- year olds for impaired central vision (18.5%), visual field loss (8.7%) and also for eye movement disorders (14.4%). Therefore, our study adds data to also support that the highest number of ocular conditions were present in the oldest stroke group. Regarding the neurological vision impairments associated with stroke because Rowe et al’s cohort was studied prospectively, the assessment battery was intentional and comprehensive, and the researchers had access to the persons’ ocular history within the year prior to stroke. Therefore, different neurological codes were identified such as: visual field loss, ocular motility problems, visual perception disorders, as compared to our study. The only the similar codes which could be a result of the stroke were diplopia (ocular motility problem) and visual field deficits [3].

Several limitations may affect the interpretation of our findings. First, this study was conducted among Medicare beneficiaries in the U.S. and may not be generalizable to stroke survivors not covered by Medicare or in settings outside of the U.S. Second, data were collected primarily for billing and administrative purposes. Certain codes may have been used more often than others since billing codes are linked to payment [17].Third, ocular diagnoses and current procedural terminology codes are usually found in the Medicare outpatient file (also known as the Carrier file), which is associated with institution care and ancillary care. We did not have the Carrier file, which requires submitting a finder file to the Research Data Assistance Center. Fourth, random and systematic coding errors can lead to under- or over-reporting of diagnoses. We were not able to determine if this data had errors since this is a retrospective review and we could not compare the claims data to electronic health records or clinical exam data. Fifth, we recognize that also because this is a retrospective review of cross sectional data, the ability to understand why there was lack of specificity, how the sequence of coding occurred, and information about the underutilization in certain codes is unknown. Also, we are not able to determine the type of visual assessments (e.g. ocular alignment and motility tests), used to determine the vision sequalae found among these stroke survivors, because this information is not listed in the claims files. Finally, as mentioned, a lack of vision screening or attention to vision in the post-acute setting may have resulted in underrepresentation of vision codes [14].

## Conclusions

This study concludes that there is ocular comorbidity in a national stroke population. Our findings suggest that the full spectrum of ICD-10 codes for characterizing eye and vision disorders may be underutilized and lack specificity in the acute stroke population. Future studies might attempt to compare ophthalmic examination results with billing codes in order to characterize the type and frequency of ocular comorbidity among stroke survivors and the sensitivity and specificity of administrative claims data for identifying them. Additionally, future work should be done to classify which vision codes commonly applied in the acute stroke setting can be translated into actionable data. Comparison to a control group without stroke in future studies may also provide important insights into factors associated with variation in the application of eye and vision codes. In addition, it would be interesting to collect data on the pathogenesis (what proportion of ischemic stroke had central retinal occlusion or branch occlusion [18]) and investigate further how the use of ICD-10 codes impacts clinical decision making, recovery, and outcomes. Through tailored interventions, the specificity of coding with ICD-10 may encourage targeted strategies to manage, prevent, or delay the burden of ocular multimorbidity. Therefore, comprehensive screening of stroke related visual impairment should occur. We suggest it is essential and relevant that hospital providers pay attention to coding practices for ocular disorders among stroke survivors in order to facilitate referrals, appropriate interventions, and optimal visual outcomes.

## Data Availability

The data that support the findings of this study are available from Medicare but restrictions apply to the availability of these data, which were used under license for the current study, and so are not publicly available.

## Abbreviations

ICD-10: Medicare International Statistical Classification of Diseases and Related Health Problems
U.S.: United States
PAC: Post Acute Care
HHA: Home Health Agency
SNF: Skilled Nursing Facility
IRF: Inpatient Rehabilitation Facility
LTAC: Long Term Assistive Care
ESRD: End Stage Renal Disease
